# Divine Emotions: On the Link Between Emotional Intelligence and Religious Belief

**DOI:** 10.1007/s10943-016-0335-3

**Published:** 2016-12-02

**Authors:** Paweł Łowicki, Marcin Zajenkowski

**Affiliations:** Faculty of Psychology, University of Warsaw, Warsaw, Poland

**Keywords:** Emotional intelligence, Religious belief, Religious orientation, Religious coping

## Abstract

There have been only few attempts to explore the relationship between emotional intelligence (EI) and religiosity. However, none of them included measures of ability EI. In two studies, we investigated the potential associations between various aspects of religious belief and ability and trait EI. In Study 1 (*N* = 240), we found that ability EI was positively associated with general level of religious belief. Study 2, conducted among Polish Christians (*N* = 159), replicated the previous result on the connection between ability EI and religion. Moreover, both trait and ability EI were negatively correlated with extrinsic religious orientation and negative religious coping. Additional analysis showed that extrinsic orientation mediated the relationship between ability EI and religiosity.

## Introduction

To date the role of emotion in religious belief and experience has been a subject of various theological, philosophical, and anthropological investigations. It has also aroused certain interest in the field of psychology, but surprisingly modest considering potential importance of this relationship (Emmons 2005). Exploring the relationship between religion and emotion might help to better understand the role of both factors in psychosocial functioning. Religion may serve as a source of certain emotion, and may influence emotional well-being (Silberman 2003; Emmons 2005). Moreover, religious beliefs and practice have been related to various processes of regulating emotions (Emmons 2005; Watts 2007; Vishkin et al. 2014). It is also claimed that religious individuals may present increased awareness of emotion, as well as greater self-control skills (Allen 1997; Geyer and Baumeister 2005). Taking it all into consideration, in this paper, we decided to focus specifically on emotional abilities and efficacy, that is emotional intelligence and their connection with various facets of religious belief.

So far, there have been few attempts to link emotional intelligence (EI) with religion. The first available report is the one by Paek (2006), who found that self-reported EI (measured with Trait meta-mood scale, TMMS) was positively correlated with certain religious behavior (e.g., church attendance and the number of religious groups attended), as well as with having an intrinsic religious orientation (an attitude treating religion as an end in itself; see Allport and Ross 1967). Personal religious orientations, within the context of EI, have also been the subject of different investigations (Liu 2010; Butt 2014). In these studies, the positive correlation between perceived emotional skills (tested with Emotional Intelligence Questionnaire) and intrinsic orientation was replicated. Moreover, having an extrinsic orientation (where religion is seen as a mean to other ends) appeared to be negatively linked to EI (Liu 2010; Butt 2014). All of these studies have shed some light on the possible association between EI and religiosity. But, at the same time, they suffer from some serious limitations as they took into consideration self-declared emotional abilities only. Thus, the aim of the current study was to examine the potential association between ability EI and religious beliefs (Study 1), as well as to deepen our understanding of the EI–religion relationship (Study 2).

There are certain similarities between religious belief and EI that let us believe that these two may be significantly connected. Only recently has religiosity been associated with the ability to mentalize or perceive minds (Gervais 2013; Waytz et al. 2010; Willard and Norenzayan 2013). This capability is considered a prerequisite for religious belief because people tend to think of deities as intentional agents with their own mental states (Gervais 2013). Simultaneously, the same mentalizing ability is related to higher EI (Barlow et al. 2010; Ferguson and Austin 2010). This perspective is further reinforced by extensive research on autism. The latter is characterized by different difficulties in social interaction associated with impaired mind-reading abilities, sometimes referred to as “mindblindness” (e.g., Baron-Cohen 1997; Baron-Cohen and Wheelwright 2004). Interestingly, the latest findings indicate that individuals with autism are also less likely to believe in a personal God and that this effect is thoroughly mediated by their ability to mentalize (Norenzayan et al. 2012). Though far from conclusive, these data on the specific ability to perceive mind provide some reasons for the potential positive correlation between religiosity and EI. Another argument worth mentioning is derived from studies on gender differences. Copious reports confirm that women generally possess higher emotional skills than men (e.g., Brackett et al. 2004; Extremera et al. 2006; Śmieja et al. 2014). When it comes to beliefs, females also tend to report greater religious involvement than males (Feltey and Poloma 1991). It is likely, then, that this regularity may also result in a significant relationship between EI and religion. Finally, one should consider the evidence on self-regulation (the process by which a person adjusts his or her behavior in pursuit of certain goals) and self-control (the ability to inhibit or alter a prepotent reaction in order to achieve some long-term goal) in association with religiosity (McCullough and Willoughby 2009). Correlational, longitudinal, and experimental research have all shown that religion can promote self-control (Geyer and Baumeister 2005; McCullough and Willoughby 2009; Rounding et al. 2012). Moreover, it has been found that religious belief has a significant impact on self-regulation by influencing people’s goals, activating self-monitoring, providing self-regulatory strength etc. (McCullough and Willoughby 2009). The ability to regulate one’s emotions and to control one’s behavior is also important definitional component of different EI concepts (Salovey and Mayer 1990; Mayer and Salovey 1997; Pérez et al. 2005). Again, this coincidence makes the EI–religion link possible.

Beside the positive association with general religiosity, EI may also predict more specific religious attitudes. The first studies on EI and religion have already demonstrated how religious orientations are related to self-reported emotional efficacy (Paek 2006; Liu 2010). Furthermore, there is also growing evidence for the connection between these religious facets and psychological adjustment. Having an intrinsic religious orientation was found to be positively correlated with self-esteem and happiness, as well as inversely correlated with anxiety, depressive symptoms, and social dysfunction (Maltby et al. 1999; Maltby and Day 2003; Lewis et al. 2005; Navara and James 2005). Moreover, research shows that higher scores on the extrinsic religious orientation scale are related to poorer psychological well-being and physical health (Maltby and Day 2003; Navara and James 2005; Maltby et al. 2010; Doane et al. 2014). The concept of EI is theoretically and empirically associated with similar indices of emotional well-being, mental health, and life satisfaction (Salovey and Mayer 1990; Schutte et al. 2002; Austin et al. 2005). Therefore, it can be presumed that EI may be positively associated with intrinsic and negatively with extrinsic orientation. Likewise, it is also possible that, through psychological adjustment, EI is linked to religious coping style (Pargament 1997). In general, positive religious coping strategy is related to various positive psychological outcomes, while negative religious coping strategy is correlated with stronger distress and worse functioning (Pargament et al. 2000, 2011). If one considers the associations between EI and adaptive coping style (Furnham et al. 2002; Matthews et al. 2004), it seems reasonable that high emotional abilities should also predict higher positive and lower negative religious coping styles.

Altogether the aforementioned findings appear to justify the following hypotheses:

### **H1**

Emotional intelligence, both trait and ability, is positively correlated with general level of religiosity.

### **H2**

Performance-based and self-perceived EI correlates positively with intrinsic religious orientation, and negatively with extrinsic religious orientation.

### **H3**

Individuals with better emotional skills present a positive religious coping style, and avoid a negative religious coping style.

## Study 1

In the first study, we tested a simple relationship between ability EI and religious belief. We referred to widely known four branch model of EI proposed by Salovey and Mayer (1990), which treats EI as a set of abilities: perception of emotions, understanding emotions, using emotions to facilitate thinking, and management of emotions. Since the aim of Study 1 was to examine the potential association between the ability model and religion, we decided to use a very general measure of religious belief that could be administered to all people, regardless of their experiences.

### Method

#### Participants

In the first study, there were 240 student participants (155 female, 83 male, 2 individuals did not report their sex) from various universities in Warsaw, Poland. The mean age was 21.08 (SD = 2.42 years, range 18–42 years).

#### Measures

##### The Test of Emotional Intelligence

The Test of Emotional Intelligence (TIE; Śmieja et al. 2014) was used to measure emotional intelligence as an ability. The scale consists of 24 item parcels, with one emotional problem situation and three possible answers in each, to which participants responded with a five-point Likert scale (from ‘very bad answer’ to ‘very good answer’). This measure, based on the theory by Salovey and Mayer (1990), aims to assess emotional intelligence understood as a set of abilities (*Perception*, *Understanding*, *Facilitation*, and *Management of emotions*). TIE has high overall reliability (*α* = .88), and lower, but satisfying, internal consistency for each subscale: *α* = .70 (*Perception*), *α* = .69 (*Understanding*), *α* = .65 (*Facilitation*), *α* = .66 (*Management*).

##### Religiosity

To assess religiosity we used a questionnaire consisting of three questions (*I believe in God*; *I believe in divine being who is involved in my life*; *There is no god or high power in the universe*). We decided on this measure because it captures a general attitude toward religious belief, and then both religious and non-religious participants can be included. The scale has been previously used by Willard and Norenzayan (2013) in their study on the cognitive basis of religion. The scale has an eight-point Likert-like scale for responses. Despite its brevity, the test has a high reliability (*α* = .85) and shows good construct validity by correlating highly with Intuitive Belief in God (Gervais and Norenzayan 2012) and the “Spiritual Well Being Scale” (Bufford et al. 1991). In the current study *α* = .86.

### Results

The conducted analysis demonstrated that religiosity was positively associated with general level of EI (see Table 1). Moreover, declared religious belief correlated positively with three out of the four specific emotional abilities (*perception, facilitation,* and *management*). The strength of each relationship is modest, but statistically significant.

**Table 1:** Means, standard deviations, and Pearson’s correlations between religiosity and emotional intelligence (*N* = 240)

	TIE—general result	TIE—perception	TIE—understanding	TIE—facilitation	TIE—management	Religiosity
Religiosity	.19**	.15*	.09	.22**	.18**	–
M	27.93	7.82	7.44	6.53	6.15	16.34
SD	5.56	1.74	1.62	1.77	1.59	6.89

* *p* < .05, ** *p* < .01

## Study 2

In the second study, we wanted to explore the relationship between EI and religiosity more deeply. We decided to include additional measures of religious experiences and therefore test only participants who identified themselves with certain religion. In particular, this study investigated Christians, the largest religious population in Poland.

### Method

#### Participants

A total of 159 adult Christians took part in the second study (104 female, 55 male) with the mean age of 23.89 (SD = 7.36 years, range 18–56 years). The following denominations participated: Roman Catholics (91.2%), Protestants (3.8%), and other Christian believers (4.4%). The structure of the sample corresponds closely with the proportion of religious affiliation in Poland (95.5% Roman Catholic) as declared in the national survey form 2011 (GUS 2013).

#### Measures

##### Religiosity, TIE

The same measures were used as described in Study 1.

##### Trait Emotional Intelligence Questionnaire—Short Form

The Trait Emotional Intelligence Questionnaire—Short Form (TEIQue-SF; Petrides and Furnham 2006; Polish adaptation by Wytykowska and Petrides 2007)—is a shortened version of a scale designed to measure global trait EI (as opposed to performance-based ability model). The 30-item test, with a seven-point Likert response scale (from ‘Completely disagree’ to ‘Completely agree’), captures not only the global result of EI, but also four emotional factors: *Well*-*being, Self*-*control, Emotionality*, and *Sociability*. The original version has high overall internal consistency (*α* = .84 for females, *α* = .89 for males).

##### Religious Orientation Scale

The Religious Orientation Scale (ROS; Allport and Ross 1967; Batson et al. 1993), in the form of the Polish version designed by Socha (1999), was used to assess individual orientations toward religion. The questionnaire consists of two scales: intrinsic (religion as an end in itself) and extrinsic (religion as a mean to some other goals) orientation. The complete test consists of 20 items with a five-point rating scale (from ‘I disagree’ to ‘I agree’). Both scales have sufficient reliability: *α* from .56 to .85 for the extrinsic orientation, *α* from .83 to .91 for the intrinsic orientation (Socha 1999).

##### The Brief RCOPE

The Brief RCOPE (Pargament et al. 2011; Polish translation by Talik 2013) is a 14-item questionnaire regarding religious coping strategies with different life stressors. The scale is intended to measure two main coping strategies: positive and negative. Participants were asked to determine how often they use certain coping strategies on four-point Likert scale (from ‘never’ to ‘always’). Internal consistency coefficients for both subscales in the present study were satisfying (*α* = .78 for negative RCOPE, and *α* = .84 for positive RCOPE).

### Results

We found that general declared religiosity was positively correlated with intrinsic religious orientation and positive religious coping style and negatively correlated with extrinsic orientation (see Table 2). These results seem consistent with the ones observed in other research (Paek 2006; Ai et al. 2010).

**Table 2:** Means, standard deviations, and Pearson’s correlations for all variables (*N* = 159)

	1	2	3	4	5	6	7	8	9	10	11	12	13	14	15
M	20.59	29.19	7.91	7.73	6.99	6.57	4.78	5.03	4.52	4.94	4.54	28.34	28.76	11.40	4.46
SD	4.58	4.32	1.60	1.44	1.39	1.14	0.79	1.26	0.95	0.89	0.97	5.55	8.15	4.76	3.72
1. Religiosity		.17*	.17*	.14^+^	.11	.08	.08	.04	.11	.13	−.04	−.25**	.66**	.50**	−.14^+^
2. TIE-gen.			.81**	.79**	.76**	.73**	.10	.07	.05	.22**	−.03	−.17*	.07	.04	−.20*
3. TIE-percep.				.56**	.45**	.41**	.04	.01	.02	.13^+^	−.04	−.13	.14	.06	−.20*
4. TIE-underst.					.42**	.45**	.05	−.01	.02	.15^+^	−.04	−.16^+^	.08	.06	−.13
5. TIE-facilit.						.51**	.12	.14^+^	.10	.19*	−.07	−.17*	.04	.03	−.11
6. TIE-manag.							.14^+^	.11	.03	.24**	.10	−.05	−.07	−.03	−.16*
7. TEIQue-gen.								.84**	.75**	.77**	.69**	−.23**	.08	.02	−.30**
8. TEIQue-w-b.									.62**	.56**	.43**	−.16*	.12	.04	−.30**
9. TEIQue-s-c.										.37**	.33**	−.13	.07	.02	−.23**
10. TEIQue-emot.											.46**	−.27**	.12	.03	−.28**
11. TEIQue-soc.												−.13	−.06	−.07	−.13
12. Extrinsic RO													−.29**	−.12	.17*
13. Intrinsic RO														.69**	−.01
14. Positive RCOPE															.18*
15. Negative RCOPE															–

^+^ *p* < .10, * *p* < .05, ** *p* < .01

The positive correlation between religiosity and the general level of ability EI from the first study was replicated. Similarly, religious belief appeared to be positively related to the perception of emotion. The associations of religiosity with other specific abilities were not statistically significant; however, understanding of emotion revealed a trend correlation (*p* < 0.10).

The extrinsic religious orientation was found to be negatively correlated with various aspects of EI. A higher score on the extrinsic scale was linked to lower emotional abilities measured by TIE (overall result and facilitation subscale). Moreover, having an extrinsic orientation correlated negatively with self-reported EI as well. Besides significant correlation with global trait EI, having an instrumental attitude toward religion was also related to two emotional factors: well-being and emotionality. Likewise, having a negative religious coping strategy proved to be negatively associated with both performance-based and self-perceived EI. Having a maladaptive coping style was related to lesser emotional skills—not only generally, but also with regard to specific skills (perception and management of emotions). Furthermore, individuals demonstrating high level of negative religious coping also assessed themselves as less emotionally intelligent by scoring significantly lower on TEIQue factors (well-being, self-control, emotionality) and on the total score.

Because both measures of EI correlated with extrinsic RO and negative coping, we decided to conduct additional regression analyses. In both tested models, the general score on TIE and TEIQue were predictors, whereas extrinsic religiosity (model 1) and negative coping (model 2) were dependent variables. In the first model, we found that trait EI predicted significantly extrinsic RO (*β* = −.22; *p* = 0.007), while ability EI was not significant (*β* = −.14; *p* = 0.083). In the second model, both EI scores significantly predicted negative coping (*β* = −.17; *p* = 0.030 for TIE; *β* = −.29; *p* < 0.001 for TEIQue).

**Fig. 1:**
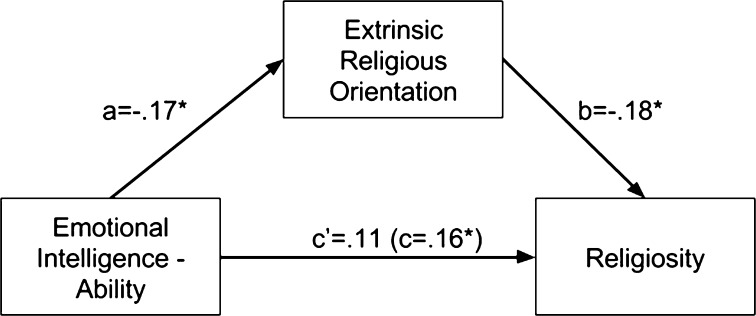
Relationships between emotional intelligence, extrinsic religious orientation, and general religiosity; *a* and *b* are direct paths, *c* is the total effect from emotional intelligence to religiosity and *c*′ is the direct path from emotional intelligence to religiosity controlling for extrinsic orientation, *p < .05

The intercorrelations between ability EI, extrinsic RO, and religiosity led us to test a mediation model. Specifically, we wanted to examine whether the link between EI and religious belief might be, to some extent, explained by person’s lower extrinsic orientation. We used the ‘PROCESS’ macro from SPSS—developed by Hayes (2015)—which tests for indirect effects by calculating (bootstrapping) confidence intervals for indirect (mediated) effects. The mediation analysis (see Fig. 1) revealed that the total effect between EI and religiosity (*β* = 0.16, *p* < 0.05) was reduced upon the inclusion of the mediator—extrinsic RO (*β* = 0.11, *p* > 0.05), indirect effect = −0.04, *p* < 0.05 (based on the bias corrected 95% confidence interval not spanning zero: lower = 0.01, upper = 0.10). Thus, the extrinsic orientation fully mediated the relationship between ability EI and general religiosity.

## Discussion

In Study 1 we focused on exploring whether religious beliefs are associated with the level of emotional abilities. The research provided the first empirical confirmation of a significant positive relationship between those two variables. Next, we conducted another study to both replicate the previous results and search for some possible mechanisms that might underlie the observed correlation.

In both conducted studies, general religiosity was positively associated with ability in EI. This indicates that more religious subjects also demonstrate higher emotional skills. However, contrary to expectations, no significant correlation was found between trait emotional intelligence (TEIQue) and religious belief. This finding seems to conflict with the hitherto data about self-perceived emotional efficacy (Liu 2010; Paek 2006). However, it is worth noting that the direction of the relationship between TEIQue results and religiosity seems predictable (generally positive), but its strength is simply very modest.

Furthermore, it was found that having an extrinsic religious orientation was related to lower ability and trait EI alike. Similarly, negative religious coping showed a negative correlation with both types of EI. This allows us to comprehend, then, that individuals who treat religion as a means and/or who use a destructive religious coping strategy, not only see themselves as less emotionally competent, but they also do have lower skills in this field. As extrinsic RO, general religiosity, and ability EI were all correlated, we decided to test a mediation model including these three variables. We supposed that the results of subjects presenting an extrinsic attitude toward religion may be in fact responsible for observed significant relationship between religious belief and emotional skills. Conducted analysis has, indeed, revealed that extrinsic religious orientation thoroughly mediated the relationship between ability emotional intelligence and declared religiosity. With reference to the studies on mind-perception (Gervais 2013; Willard and Norenzayan 2013), this can be interpreted to mean that emotionally intelligent individuals may also present a high level of mentalizing ability, and that, as a result, they are capable of genuinely involving themselves in religion. In contrast, people with lower emotional skills may also lack certain mind-reading skills, and thus if they do become religious at all, it is likely going to take the form of extrinsic orientation, which treats faith as a means to certain goals. These results are consistent with the findings showing that people with high levels of certain antisocial personality traits (i.e., psychopathy and Machiavellianism) who are known to have poor emotional and social skills exhibit low religiosity (Łowicki and Zajenkowski in press). One can then presume that people with low EI are somehow unable to develop a sincere, disinterested belief in God. However, they may nevertheless declare to be religious. This could be because, for example, they were raised in a religious tradition, or because they want to achieve something through their religious commitment. This intuition seems even more adequate when some items from the extrinsic religious orientation scale are considered (e.g., I pray chiefly because I have been taught to pray; a primary reason for my interest in religion is that my church is a congenial social activity; one reason for being a church member is that such membership helps to establish a person in the community). Moreover, emotionally intelligent individuals can also regulate their emotions on their own, while less intelligent people may use religion in order to bring about similar effects in themselves (e.g., the primary purpose of prayer is to gain relief and protection; the purpose of prayer is to secure a happy and peaceful life).

Although we have tested a mediational model, the existence of causal relationships and their potential direction still remains an open problem. To determine it definitely, an experimental research should be carried out. Nevertheless, there are some indirect cues that suggest that it is EI that is affecting religiosity. For instance, with regard to academic intelligence, we can claim that analytic style of thinking promotes lower level of religiosity (Zuckerman et al. 2013; Gervais and Norenzayan 2012). What is more, an ability to perceive and attribute minds to other beings, it is argued, helps to enable the development of religious beliefs (Gervais 2013). By analogy to these suggestions, we can suppose that certain level of EI is also a prerequisite for becoming truly engaged in religion. Obviously, these are simply reasonable conjectures; at this stage, we cannot rule out that reverse causality is possible in this context.

Interestingly, both “positive” aspects of religiosity—intrinsic orientation and positive coping—appeared to be independent of any emotional factors. This result is inconsistent with previous findings (Paek 2006; Liu 2010; Butt 2014) and may be explained, at least to some extent, by cultural differences. The sample in our study was predominantly Roman Catholic (which is the dominant religion in Poland), which stands in contrast to the different denominations that made up the samples in other studies (Paek 2006). It is generally admitted, for instance, that Protestantism is related to more individualistic and more personal attitude toward religion, while Catholicism emphasizes participation in ceremonies and maintenance of tradition—as studies have shown this may result in significant differences in the religious orientations of these denominations (see Park et al. 1990). Taking all this into consideration, it is worth noting that EI is, consequently, correlated with negative subdimensions of religious belief. Thus, it seems possible that high EI might be a factor that protects against maladaptive religiosity. This conclusion seems to cohere with the extensive data on the positive psychological and social outcomes associated with EI (e.g., Schutte et al. 2002; Austin et al. 2005; Gallagher and Vella-Brodrick 2008).

The current investigation contributes substantially to better understanding of the relationship between EI and religiosity. However, the study has certain limitations. Specifically, we did not include some control variables which may potentially influence the observed results. While trait EI is connected with personality, ability EI is widely associated with intelligence (Petrides and Furnham 2006; Śmieja et al. 2014). Therefore, in the future investigation, it is necessary to verify the role of these characteristics in EI–religion relationship. Moreover, controlling for the level of mentalizing ability might be also revealing, as would an experimental study that primed for either religiosity or emotional content. Furthermore, there have also been a few attempts to link EI with the efficiency of emotional information processing (e.g., Austin 2005). Thus, one may wonder whether religiosity, similarly to EI, is predictive of the performance on simple emotional tasks. The research could also be extended to other denominations and religions to confirm its validity.
